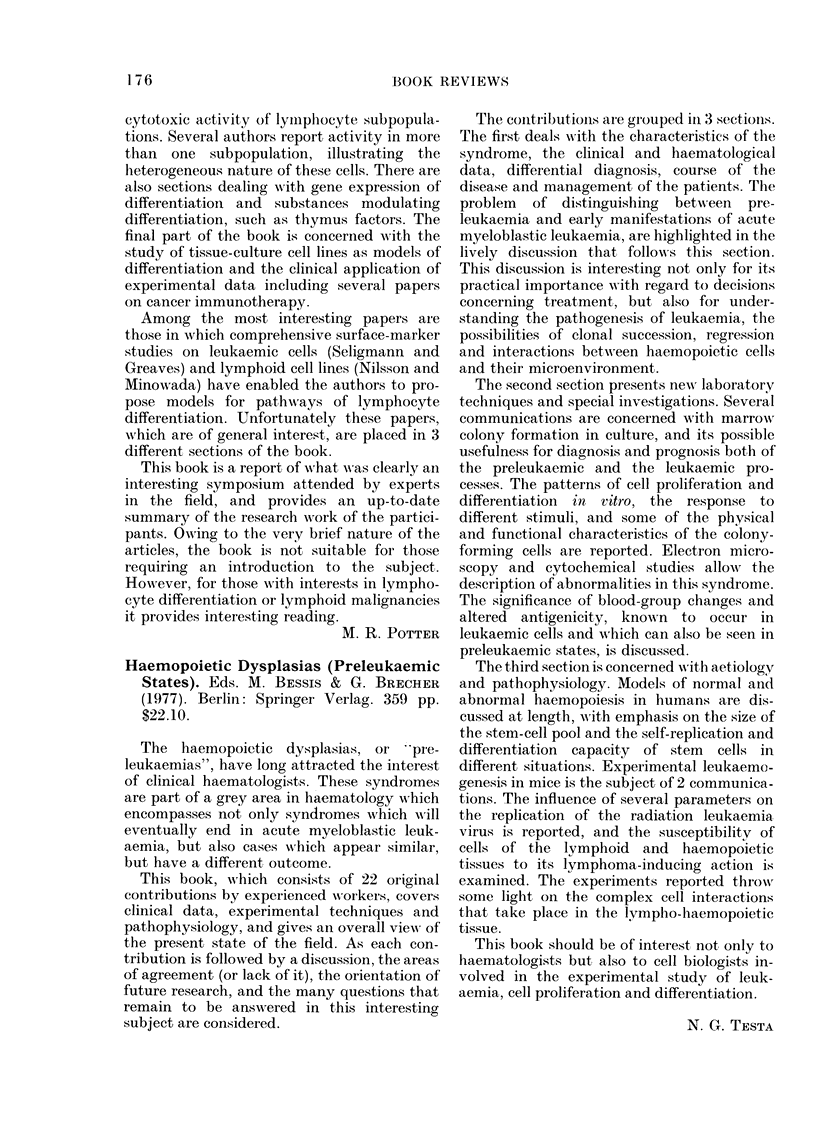# Haemopoietic Dysplasias (Preleukaemic States)

**Published:** 1979-07

**Authors:** N. G. Testa


**Haemopoietic Dysplasias (Preleukaemic States).** Eds. M. BESSIS & G. BRECHER (1977). Berlin: Springer Verlag. 359 pp. $22.10.

The haemopoietic dysplasias, or "preleukaemias", have long attracted the interest of clinical haematologists. These syndromes are part of a grey area in haematology which encompasses not only syndromes which will eventually end in acute myeloblastic leukaemia, but also cases which appear similar, but have a different outcome.

This book, which consists of 22 original contributions by experienced workers, covers clinical data, experimental techniques and pathophysiology, and gives an overall view of the present state of the field. As each contribution is followed by a discussion, the areas of agreement (or lack of it), the orientation of future research, and the many questions that remain to be answered in this interesting subject are considered.

The contributions are grouped in 3 sections. The first deals with the characteristics of the syndrome, the clinical and haematological data, differential diagnosis, course of the disease and management of the patients. The problem of distinguishing between preleukaemia and early manifestations of acute myeloblastic leukaemia, are highlighted in the lively discussion that follows this section. This discussion is interesting not only for its practical importance with regard to decisions concerning treatment, but also for understanding the pathogenesis of leukaemia, the possibilities of clonal succession, regression and interactions between haemopoietic cells and their microenvironment.

The second section presents new laboratory techniques and special investigations. Several communications are concerned with marrow colony formation in culture, and its possible usefulness for diagnosis and prognosis both of the preleukaemic and the leukaemic processes. The patterns of cell proliferation and differentiation *in vitro*, the response to different stimuli, and some of the physical and functional characteristics of the colony-forming cells are reported. Electron microscopy and cytochemical studies allow the description of abnormalities in this syndrome. The significance of blood-group changes and altered antigenicity, known to occur in leukaemic cells and which can also be seen in preleukaemic states, is discussed.

The third section is concerned with aetiology and pathophysiology. Models of normal and abnormal haemopoiesis in humans are discussed at length, with emphasis on the size of the stem-cell pool and the self-replication and differentiation capacity of stem cells in different situations. Experimental leukaemogenesis in mice is the subject of 2 communications. The influence of several parameters on the replication of the radiation leukaemia virus is reported, and the susceptibility of cells of the lymphoid and haemopoietic tissues to its lymphoma-inducing action is examined. The experiments reported throw some light on the complex cell interactions that take place in the lympho-haemopoietic tissue.

This book should be of interest not only to haematologists but also to cell biologists involved in the experimental study of leukaemia, cell proliferation and differentiation.

N. G. TESTA